# Targeting angiogenesis for fracture nonunion treatment in inflammatory disease

**DOI:** 10.1038/s41413-021-00150-4

**Published:** 2021-06-07

**Authors:** Cuicui Wang, Jun Ying, Xiaolei Nie, Tianhong Zhou, Ding Xiao, Gaurav Swarnkar, Yousef Abu-Amer, Jianjun Guan, Jie Shen

**Affiliations:** 1 Department of Orthopaedic Surgery, School of Medicine, Washington University, St. Louis, MO USA; 2 Department of Orthopaedic Surgery, the First Affiliated Hospital of Zhejiang Chinese Medical University, Hangzhou, China; 3 Institute of Orthopaedics and Traumatology, the First Affiliated Hospital of Zhejiang Chinese Medical University, Hangzhou, China; 4 Department of Mechanical Engineering & Materials Science, School of Engineering, Washington University, St. Louis, MO USA; 5 Shriners Hospital for Children, St. Louis, MO USA

**Keywords:** Bone, Diseases

## Abstract

Atrophic fracture nonunion poses a significant clinical problem with limited therapeutic interventions. In this study, we developed a unique nonunion model with high clinical relevance using serum transfer-induced rheumatoid arthritis (RA). Arthritic mice displayed fracture nonunion with the absence of fracture callus, diminished angiogenesis and fibrotic scar tissue formation leading to the failure of biomechanical properties, representing the major manifestations of atrophic nonunion in the clinic. Mechanistically, we demonstrated that the angiogenesis defect observed in RA mice was due to the downregulation of SPP1 and CXCL12 in chondrocytes, as evidenced by the restoration of angiogenesis upon SPP1 and CXCL12 treatment in vitro. In this regard, we developed a biodegradable scaffold loaded with SPP1 and CXCL12, which displayed a beneficial effect on angiogenesis and fracture repair in mice despite the presence of inflammation. Hence, these findings strongly suggest that the sustained release of SPP1 and CXCL12 represents an effective therapeutic approach to treat impaired angiogenesis and fracture nonunion under inflammatory conditions.

## Introduction

Approximately ten percent of the 16 million fractures that occur annually in the United States do not progress to timely union and exhibit prolonged healing or nonunion.^1,2^ There are two distinct types of fracture nonunion characterized by different radiographical observations and biological properties, atrophic and hypertrophic nonunion.^3^ The development of hypertrophic nonunion is primarily due to inadequate mechanical stability that leads to excessive fracture callus formation; therefore, appropriate mobilization and fixation at the fracture site are usually used in the clinic to achieve a successful outcome in hypertrophic nonunion patients.^4^ In contrast, atrophic nonunion is characterized by limited fracture callus with poor tissue revascularization, likely secondary to the perturbation of normal biological cues. Treatment of atrophic nonunion usually involves complex clinical intervention practices and often requires multiple surgeries. Thus, atrophic nonunion results in significant patient disability and increased cost to the health care system.^5^ While bone graft surgeries, mechanical stimulation devices, and therapies using growth factors and stem cells have been developed, the effective management of atrophic nonunion is limited, and atrophic nonunion remains a major clinical challenge for orthopedic surgeons.

A major population affected by atrophic nonunion is patients with inflammatory conditions, e.g., elderly patients, individuals who smoke, and diabetic or rheumatoid arthritis (RA) patients.^6–8^ In these patients, the fracture risk is increased due to poor bone quality,^7,9–12^ highlighting the potential deleterious role of chronic systemic inflammation in fracture repair. This can be observed in preclinical models. Indeed, TNFα-transgenic mice showed impaired bone quality, including reduced cortical thickness, leading to decreased fracture toughness.^13^ In addition, experiments using a mouse cortical defect model showed that bone regeneration was also significantly reduced in RA mice.^14^ Studies from patients and rodents have extensively reported that chronic systemic inflammation activates the canonical NF-κB pathway, resulting in the elevated expression of IL-1β, TNFα, and other cytokines,^12,15–18^ impairing the fracture repair process at least partially by negatively affecting angiogenesis.^19–22^ Although pharmacological anticytokine therapies have been developed and are highly effective in RA patients,^23–26^ the impact of these agents on fracture healing in patients with inflammatory arthritis is not known. Preclinical animal studies showed that the TNFα inhibitor infliximab had a positive effect on the restoration of callus formation and biomechanical properties of fractured bone in wild-type rats under chronic inflammatory conditions.^27^ In contrast, a human cohort study in ankylosing spondylitis patients demonstrated the negative long-term effect of TNFα inhibitor treatment on fracture healing.^28^ Therefore, there is an urgent need to develop molecular therapies for fracture nonunion, especially for older patients with chronic inflammatory diseases. In this study, we show that serum transfer-induced RA (also known as K/BxN) mice develop atrophic nonunion, with the absence of fracture callus, and coincident diminished angiogenesis. Despite the large amount of evidence on the role of the NF-κB pathway in fracture studies, the mechanisms by which pathologic inflammation adversely affects angiogenesis during fracture healing are largely unknown. Here, we used this novel RA nonunion model to study the cellular and molecular basis of the negative impact of inflammation on angiogenesis.

During normal fracture repair, chondrocytes and osteoblasts are the primary cell types that secrete angiogenic factors, recruiting endothelial cells and facilitating angiogenesis and vasculogenesis.^29–33^ As a key initial step, the re-established vascular network brings oxygen and nutrients to facilitate bone regeneration in addition to osteoprogenitors, osteoblasts and other cells, which are necessary for callus maturation and bone formation. However, under inflammatory conditions, insufficient revascularization^34,35^ occurs, particularly leading to atrophic nonunion.^36–40^ Through an unbiased in vitro screen of angiogenic factors, we found that secreted phosphoprotein 1 (SPP1) and C-X-C motif chemokine ligand 12 (CXCL12) are the two factors downregulated to the greatest extent by IL-1β treatment, suggesting SPP1 and CXCL12 as potential targets of inflammation in chondrocytes. Both SPP1^41–44^ and CXCL12^45–48^ are highly expressed in chondrocytes and osteoblasts during fracture repair, and recent rodent studies suggest that SPP1 and CXCL12 contribute to fracture healing through the improvement of neovascularization.^49,50^ To target the angiogenesis process, we developed biodegradable scaffolds capable of continuously releasing SPP1 and CXCL12 locally at the fracture site. Strikingly, the sustained delivery of SPP1 and CXCL12 accelerated fracture union in RA mice and restored biomechanical properties, highlighting this approach as a potential therapeutic strategy to treat atrophic fracture nonunion in patients with inflammatory diseases.

## Results

### Elevated inflammation led to fracture nonunion in RA mice

**Fig. 1:**
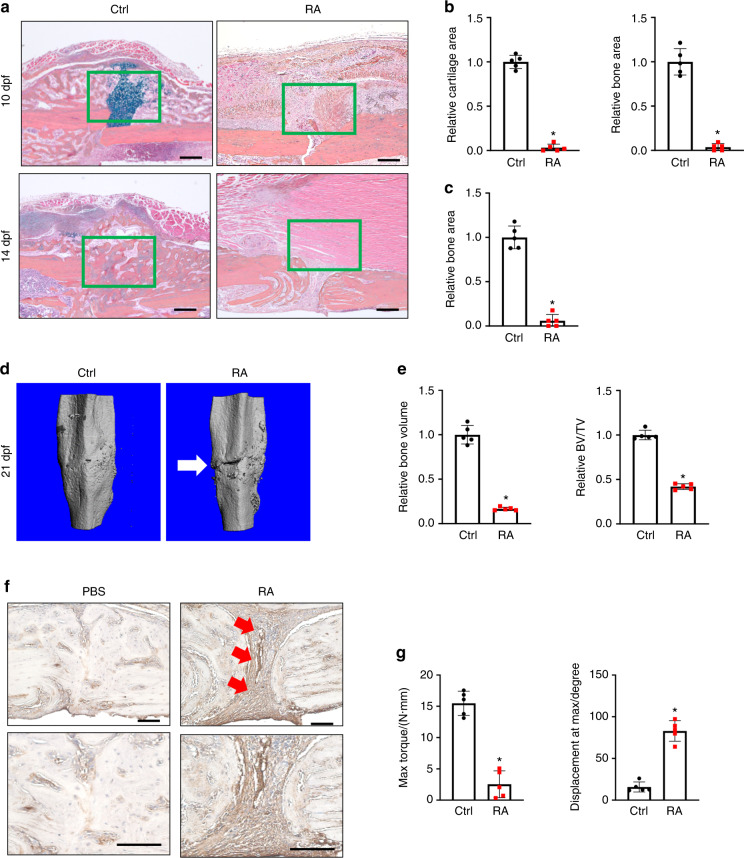
Systemic inflammation-induced fracture nonunion in RA mice. **a** Representative ABH/OG staining of fracture callus sections from control and RA mice at 10 and 14 dpf (*n* = 5). Histomorphometric quantification of cartilage and bone area was performed on (**b**) 10 dpf and (**c**) 14 dpf fracture callus sections from the control and RA mice (*n* = 5). The results were normalized to the controls. *P < 0.05 compared with control by Student’s *t* test. **d** MicroCT assessment of mineralized bone within the fracture callus at 21 dpf from the control and RA mice (*n* = 5). **e** Quantification of bony callus volume and relative BV/TV ratio based on microCT assessment of 21 dpf control and RA fracture calluses. All results were normalized to controls. *P < 0.05 compared with control by Student’s *t* test. **f** Immunohistochemical staining of 21 dpf fracture calluses from control and RA mice for COL3A1. **g** Biomechanical torsion testing of the control and RA fractures at 28 dpf (*n* = 5). Max torque and displacement at max were quantified. All results were normalized to the controls. *P < 0.05 compared with control by Student’s *t-*test. Scale bar, 200 μm

The rising global prevalence of inflammatory diseases, especially RA,^51–53^ is associated with debilitating comorbidities and clinical complications, including delayed fracture union and nonunion.^8,54,55^ To examine the effect of inflammation/RA on fracture repair, we generated RA mice via repeated intraperitoneal (i.p.) K/BxN serum administration every 5 days to maintain systemic inflammation. Given that the transcription factor NF-κB is the principal mediator and agent of inflammatory responses, we examined its activity using NF-κB/RelA-Luc reporter mice subjected to RA conditions. Consistent with the expected increase in inflammation, we confirmed that the RA mice displayed significantly higher luciferase activity, especially in the limbs, compared to that in the control group (Fig. S1A). Importantly, inflammation was elevated, peaking at 10 days after serum administration, and this increase was maintained for at least 15 days (Fig. S1B). Clinical reports and rodent studies have shown that elevated inflammatory cytokines are the major factors that contribute to RA-related comorbidities. Indeed, we showed that mRNA expression levels of the inflammatory cytokines *Il1β*, *Il6*, *Il10,* and *Tnfα* were significantly elevated in the fracture calluses of the RA mice (Fig. S2). Importantly, compared to the control mice, the RA mice developed impaired fracture repair with diminished cartilaginous and bony callus formation, as reflected by histological analyses at 10 and 14 days post fracture (dpf) tibiae (Fig. 1a). Consistent with the histology results, quantitative histomorphometry confirmed a minimal cartilage template and newly formed woven bone in the RA callus at 10 and 14 dpf (Fig. 1b, c). We then performed microCT to further examine the mineralized callus in the control and RA mice. We found a gradual increase in new bone formation around the fracture area by 14 dpf in the control mice. The fracture gap in the control mice had closed at 21 dpf, with robust bone formation and complete healing. In contrast, the fracture gap in the RA mice persisted until 21 dpf, as shown by microCT, with significantly reduced newly formed bone tissue and bone volume over total volume (BV/TV) ratio (Figs. 1d, e; S3). Notably, collagen III-positive fibrotic tissue was present in the center of the RA fracture callus instead of woven bone tissue in the control fracture, suggesting an atrophic nonunion fracture in the RA mice (Fig. 1f). Finally, we performed torsion testing to evaluate the mechanical properties of the tibia at 28 dpf as the ultimate indicator of fracture repair outcome in the control and RA mice. Compared to the fractured tibiae of control mice, the fractured tibiae of RA mice displayed significantly lower maximum torque (77% reduction) with a larger displacement angle, indicating poor bone strength and rigidity of newly formed bone in the RA mice (Fig. 1g). Altogether, these data confirmed that under RA conditions, mice displayed systemic inflammation mediated by cytokines and developed fracture nonunion.

### Inflammation reduced the expression of angiogenic factors and impaired angiogenesis in mice

**Fig. 2:**
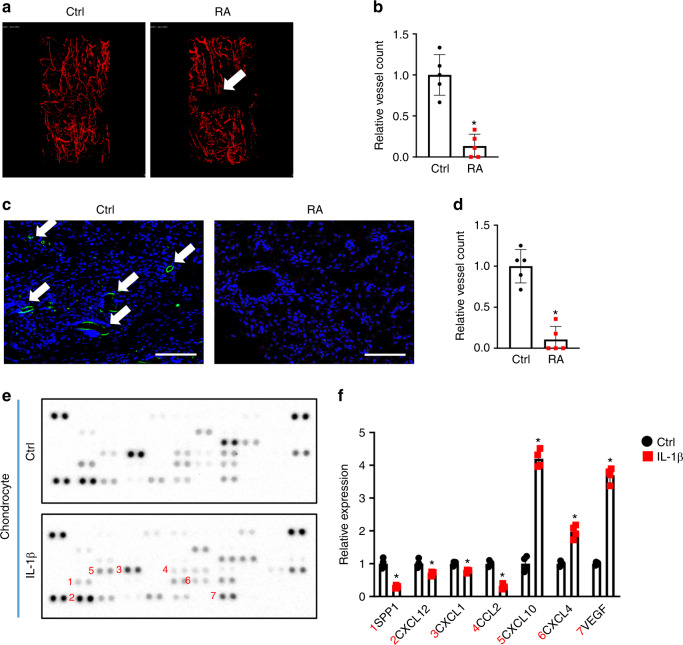
Systemic inflammation led to impaired angiogenesis. **a** MicroCT assessment of newly formed vessels within 10 dpf fracture calluses from control and RA mice. **b** Quantification of vessel counts in 10 dpf control and RA fracture calluses (*n* = 5) based on microCT assessment. The results were normalized to the controls. *P < 0.05 compared with control by Student’s *t* test. **c** Immunohistochemical staining for endomucin in 10 dpf fracture calluses from the control and RA mice. **d** Quantification of vessel counts in 10 dpf control and RA fracture calluses (*n* = 5) based on the immunohistochemical assessment. The results were normalized to the controls. *P < 0.05 compared with control by Student’s *t* test. **e** Representative images of an angiogenesis proteasome array from the culture medium of chondrocytes following vehicle and IL-1β treatment. **f** Quantification of blot intensity from the angiogenesis proteasome array (*n* = 4). All results were normalized to the controls. *P < 0.05 compared with control by Student’s *t* test. Scale bar, 200 μm

Clinical studies and rodent models have established that defects in vascularization coincide with fracture nonunion, especially under inflammatory conditions, such as those in RA. Consistent with these reports, we observed that the control mice formed blood vessels at 10 dpf, the peak angiogenic time point in murine fracture healing. In contrast, callus tissues from the RA mice showed diminished angiogenesis and poor angiogenic connectivity at 10 dpf (Fig. 2a). Quantification confirmed significantly fewer blood vessels in the fractured calluses of RA mice at 10 dpf (Fig. 2b). We also performed immunohistochemistry (IHC) to detect endomucin-positive blood vessels in the fractured calluses. Similar to the angiography results, IHC revealed almost nondetectable blood vessels in the fractured calluses of RA mice, but abundant blood vessels were observed in the fractured calluses of control mice at 10 dpf (Fig. 2c, d). These data indicate that systemic inflammation had adverse effects on the expression of angiogenic factors, leading to reduced angiogenesis during fracture repair. Since chondrocytes and osteoblasts^31–33,56–59^ are the primary cell types that secrete angiogenic factors in the fracture callus to stimulate blood vessel formation,^36,60,61^ we isolated primary chondrocytes and osteoblasts and treated them with IL-1β in vitro for 72 h to examine the effect of inflammation on the production of angiogenic factors by these cells. A protein array (53 angiogenic factors) was used to determine the presence of angiogenic mediators in the culture supernatant from chondrocytes and osteoblasts. Interestingly, we noticed that inflammation significantly reduced expression of the angiogenic factors SPP1, CXCL12, C-X-C motif chemokine ligand 1 (CXCL1), and C-C motif chemokine ligand 2 in chondrocytes but induced the expression of C-X-C motif chemokine ligand 4 (CXCL4), C-X-C motif chemokine ligand 10 (CXCL10) and vascular endothelial growth factor (VEGF) (Fig. 2e, f). Surprisingly, the expression of angiogenic factors in osteoblasts was minimally altered by IL-1β treatment (Fig. S4A). Even the expression of SPP1, VEGF, platelet-derived growth factor, and placental growth factor 2 was significantly increased under IL-1β treatment in primary osteoblasts (Fig. S4B). These data highlight the key role of chondrocytes in the angiogenesis defects observed in the RA mice during fracture nonunion. Since other inflammatory cytokines, such as IL-6 and TNFα, were shown to be upregulated in the fractured callus in RA (Fig. S2), we also treated primary chondrocytes with IL-6 and TNFα. Similar to the effect of IL-1β, treatment with IL-6 and TNFα also reduced the protein expression of SPP1 and CXCL12 in chondrocytes (Fig. S5).

### SPP1 and CXCL12 restored angiogenesis in inflammation in vitro

**Fig. 3:**
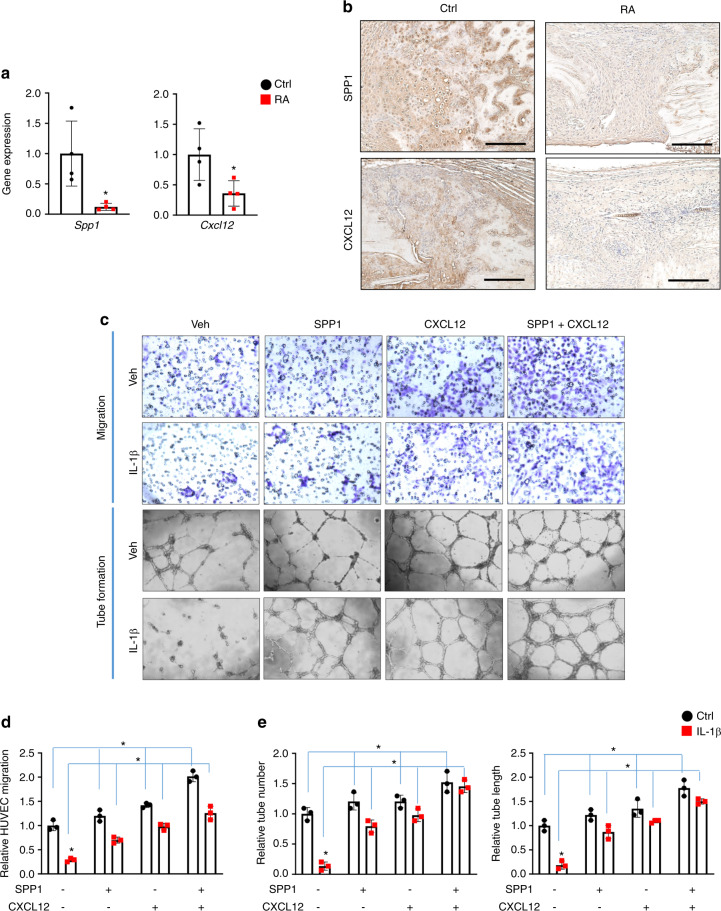
SPP1 and CXCL12 restored angiogenesis under inflammation in vitro. **a** Real-time qPCR analyses were performed to determine the relative expression of *Spp1* and *Cxcl12* in 10 dpf fracture calluses from control and RA mice (*n* = 4). The mRNA levels were normalized to that of *Actb* and then normalized to the control group. *P < 0.05 compared with control by Student’s *t* test. **b** Immunohistochemical staining of 10 dpf fracture calluses from control and RA mice for SPP1 and CXCL12. **c** Representative images from HUVEC migration and tube formation assays using culture medium from vehicle- and IL-1β-treated chondrocytes supplemented with SPP1 and CXCL12, respectively. Quantification of (**d**) HUVEC migration as well as (**e**) tube number and tube length (*n* = 3). All results were normalized to the controls. *P < 0.05 compared with control by two-way ANOVA. Scale bar, 200 μm

Since SPP1, the most abundantly expressed factor in chondrocytes, was the factor most significantly reduced by IL-1β and CXCL12, we then focused on these two angiogenic factors and examined whether SPP1 and CXCL12 are the downstream targets of inflammation that mediate angiogenic defects. On the one hand, we confirmed that, consistent with the protein expression, *Spp1* and *Cxcl12* gene expression was also decreased in the fractured calluses of RA mice at 10 dpf compared to those of control mice (Fig. 3a). Furthermore, IHC analysis of fractured tissue at 10 dpf revealed abundant expression of SPP1 and CXCL12 in the fractured calluses of control mice but almost nondetectable expression of SPP1 and CXCL12 in the fractured calluses of RA mice (Fig. 3b), confirming the downregulation of SPP1 and CXCL12 mediated by inflammation in the context of fracture repair under RA conditions. To confirm the physiological effect of inflammation on chondrocyte-mediated regulation of angiogenesis, we examined the in vitro angiogenesis of human umbilical vein endothelial cells (HUVECs) using culture medium supernatants collected from primary chondrocytes treated with vehicle or IL-1β (Fig. 3c–e). Consistent with our in vivo findings on angiogenesis, the control culture supernatants from vehicle-treated chondrocytes exhibited robust cell migration as well as an abundance of well-developed vessel tubes in vitro. In contrast, significant reductions in HUVEC migration, tube number, and tube length were observed in the presence of culture supernatant from IL-1β-treated chondrocytes. This was not related to the continued presence of IL-1β in the supernatant, since our angiogenic factor protein array confirmed that there was no IL-1β left in the culture medium after 72 h of IL-1β treatment (Fig. 2e). In addition, compared to culture medium from vehicle-treated chondrocytes, culture medium from IL-1β-treated chondrocytes did not clearly alter HUVEC proliferation or apoptosis (Fig. S6), suggesting that the decreased angiogenic capacity of the culture medium was at least partially attributed to reduced SPP1 and CXCL12 but not dysfunction of the HUVECs themselves. Therefore, based on our previous studies, we applied 500 ng·mL^−1^ SPP1 and 100 ng·mL^−1^ CXCL12 to the chondrocyte culture medium and performed HUVEC angiogenesis assays. As expected, the administration of SPP1 and CXCL12 individually or in combination to the culture medium from vehicle-treated chondrocytes resulted in the induction of HUVEC migration and tube formation. More importantly, exogenous SPP1 and CXCL12 administration restored angiogenic capacity, as reflected by increased cell migration, tube number, and tube length with culture medium from IL-1β-treated chondrocytes. We also noticed that the combined administration of SPP1 and CXCL12 had the most profound restorative effect on in vitro HUVEC angiogenesis. These data, together with the murine RA fracture nonunion model, identify reductions in SPP1 and CXCL12 as the molecular basis for the negative impact of inflammation on angiogenesis and present SPP1 and CXCL12 as potential therapeutic approaches to treat fracture nonunion.

### Delivery of SPP1 and CXCL12 via a biodegradable scaffold

**Fig. 4:**
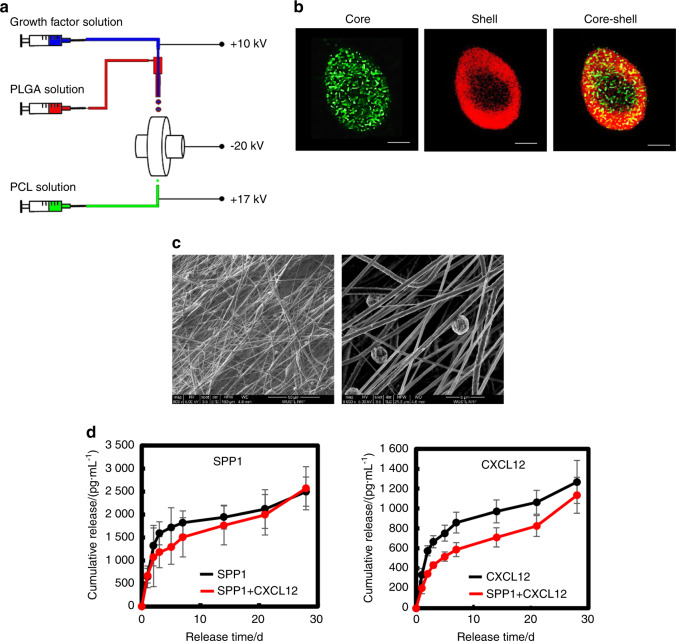
PCL scaffolds gradually released SPP1 and CXCL12 in vitro. **a** Schematic illustration of the simultaneous electrospinning and electrospraying system for PCL scaffold fabrication. **b** Representative confocal images of the PLGA microspheres encapsulated with growth factors. PLGA: red; growth factor: green. **c** Representative SEM images of the PCL scaffold. **d** Profiles of SPP1 and CXCL12 release from PCL scaffolds loaded with SPP1 and CXCL12 (*n* = 5). Scale bar, 200 μm

To promote angiogenesis at fracture sites while avoiding excessive angiogenesis in other tissues, we designed a scaffold made of polycaprolactone (PCL), a US Food and Drug Administration (FDA)-approved biodegradable polymer for tissue engineering applications, to deliver SPP1 and CXCL12 locally.^62^ We used our unique electrospinning and electrospraying system^63^ to fabricate PCL scaffolds, i.e., by simultaneously electrospinning PCL fibers and electrospraying poly(lactide-co-glycolide) (PLGA) microspheres loaded with SPP1 (100 μg·mL^−1^) and/or CXCL12 (20 μg·mL^−1^) (Fig. 4a). To demonstrate the core–shell structured microspheres, we mixed a PLGA solution with rhodamine B and simultaneously mixed growth factor solution with Hoechst. The fluorescent images shown in Fig. 4b confirmed that the microspheres assumed a core–shell structure with PLGA as the shell and SPP1 or CXCL12 as the core (Fig. 4b). The scaffold structure and microsphere distribution were characterized by scanning electron microscopy (SEM). The PCL scaffold was formed by interlaced fibers, and microspheres with a diameter of ~5 μm were distributed uniformly in the scaffolds (Fig. 4c). The scaffolds were strong, with a tensile strength of 28.8 MPa and Young’s modulus of 111.5 MPa (Fig. S7). Finally, we examined the release profiles of SPP1 and CXCL12 in vitro over a 4-week period. According to the ELISAs of the collected medium, SPP1 and CXCL12 exhibited a two-stage release pattern (Fig. 4d). Both displayed burst release in the first 3 days, followed by controlled slow release until day 28. Furthermore, the release of SPP1 and CXCL12 from the scaffold loaded with both SPP1 and CXCL12 was slower than that from the scaffold loaded with each individual growth factor (Fig. 4d).

### Release of SPP1 and CXCL12 promotes angiogenesis under inflammatory conditions

**Fig. 5:**
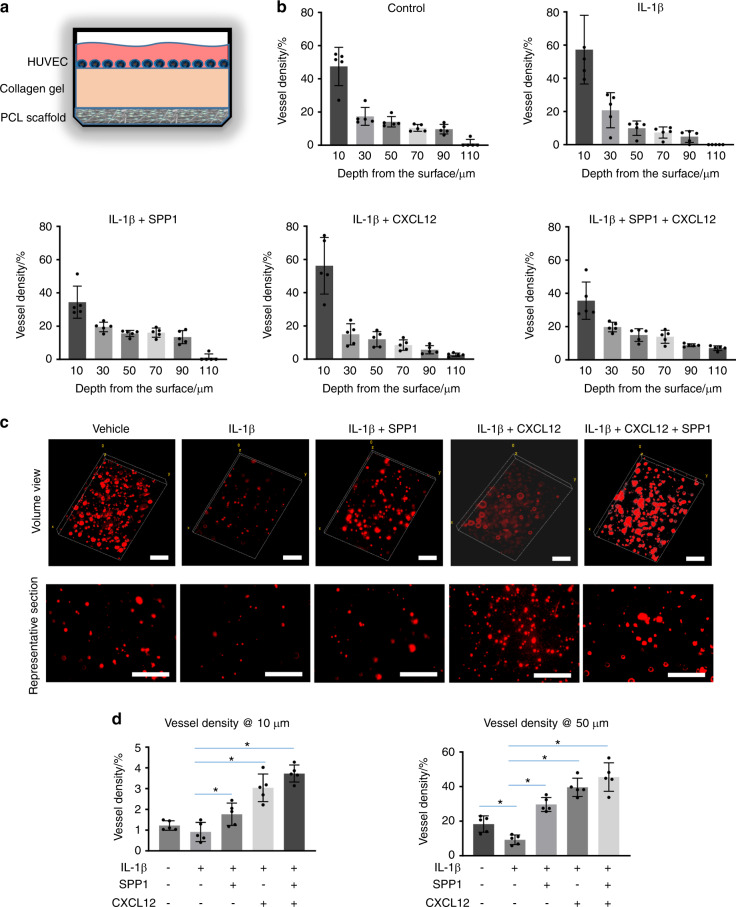
The release of SPP1 and CXCL12 promoted angiogenesis under inflammatory conditions. **a** Schematic illustration of the collagen construct that was used to create the 3D cell culture environment to examine the impact of released growth factors on HUVEC migration and tube formation. **b** Quantification of HUVEC numbers at different depths in collagen gel (*n* = 5). All results were normalized to the controls. *P < 0.05 compared with control by two-way ANOVA. **c** Representative images of HUVEC lumen formation in collagen gels. **d** Quantification of lumen density at different depths in the collagen gel (*n* = 5). *P < 0.05 compared with control by two-way ANOVA. Scale bar, 200 μm

Since the RA mice displayed a systemic inflammatory environment, particularly the increased expression of inflammatory cytokines identified in the fracture sites (Fig. S2), we examined whether the SPP1 and CXCL12 released from the scaffolds could promote HUVEC angiogenic capacity under inflammatory conditions. We mimicked the scenario in which the scaffold is implanted at the fracture site. We placed collagen gel on top of PCL scaffolds loaded with SPP1 and/or CXCL12 and then cultured CM-Dil dye-labeled HUVECs on the collagen gel under control or inflammatory conditions (Fig. 5a). The migration of HUVECs on the collagen gel was then examined by confocal microscopy. As expected, IL-1β treatment significantly reduced HUVEC migration, particularly in the deep zone from 50 to 110 μm from the top surface (Fig. 5b). However, growth factors substantially promoted migration under IL-1β treatment (Fig. 5b). The SPP1 released from the scaffold loaded with SPP1 alone promoted HUVEC migration to a greater extent than the CXCL12 released from the scaffold loaded with CXCL12 alone. The greatest cell migration was found in the group with both SPP1 and CXCL12 release, especially in the deep zone up to 110 μm from the top surface (Fig. 5b). In addition to cell migration, we examined tube formation based on lumens formed in the collagen gel, with a particular focus on the area containing the majority of the HUVECs. IL-1β treatment significantly reduced tube formation, as reflected by decreased lumen formation in the collagen gel in the top surface (10 μm) and deep zone (50 μm), although no difference in HUVEC migration was observed on the surface under IL-1β treatment. Importantly, lumen formation was restored in the groups in which scaffolds were loaded with SPP1 and CXCL12, even under inflammatory conditions. The lumen density in the group with only CXCL12 release was significantly higher than that in the group with only SPP1 release. The group with both SPP1 and CXCL12 release had a significantly greater lumen density than the group with CXCL12 release alone both on the surface and in the deep zone (Fig. 5c, d). Altogether, these data confirmed that SPP1 and CXCL12 were gradually released over 4 weeks from the scaffolds, which could potentially benefit angiogenesis and inflammatory bone fracture healing.

### Controlled release of SPP1 and CXCL12 restored angiogenesis and fracture union in RA mice

**Fig. 6:**
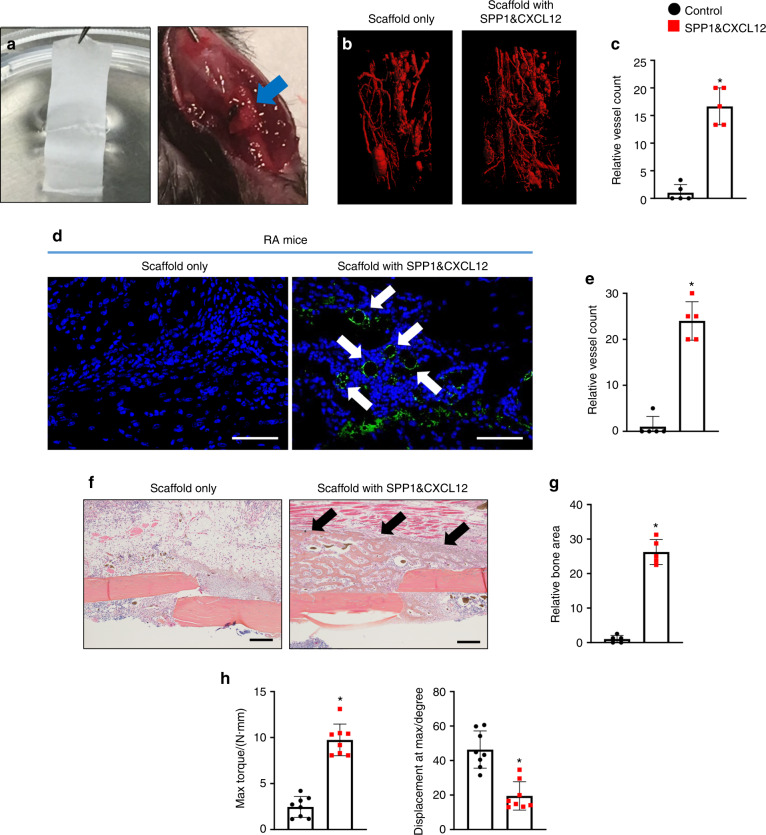
The controlled release of SPP1 and CXCL12 restored angiogenesis and fracture nonunion in RA mice. **a** PCL scaffold with or without SPP1 and CXCL12 was applied to the fractured bone in RA mice. **b** MicroCT assessment of newly formed vessels within fracture calluses from RA mice treated with scaffolds with or without SPP1 and CXCL12 (*n* = 5) at 10 dpf. **c** Quantification of the vessels in RA fracture calluses (*n* = 5) at 10 dpf based on microCT assessment. The results were normalized to the scaffold only group. *P < 0.05 compared with control by Student’s *t-*test. **d** Immunohistochemical staining for endomucin in fracture calluses from RA mice at 10 dpf. **e** Quantification of the vessels in RA fracture calluses at 10 dpf (*n* = 5) based on the immunohistochemical assessment. The results were normalized to the scaffold only group. *P < 0.05 compared with control by Student’s *t* test. **f** Representa*t*ive ABH/OG staining of fracture callus sections from RA mice treated with scaffold with or without SPP1 and CXCL12 at 10 dpf (*n* = 5). **g** Histomorphometric quantification of bone area was performed on 10 dpf fracture callus sections from RA mice treated with scaffolds with or without SPP1 and CXCL12 (*n* = 5). The results were normalized to the scaffold group. **h** Biomechanical torsion testing of RA fractures treated with scaffold with or without SPP1 and CXCL12 at 28 dpf (*n* = 8). Max torque and displacement at max were quantified. All results were normalized to the controls. *P < 0.05 compared with control by Student’s *t* test. Scale bar, 200 μm

With the observation of the bioactivity of PCL scaffolds in vitro, we applied the scaffolds with SPP1 (100 μg·mL^−1^) and/or CXCL12 (20 μg·mL^−1^) to treat fracture nonunion in RA mice. A 2-mm scaffold was wrapped around the fractured bone immediately after the fracture procedure (Fig. 6a). Vascular structure and the fracture healing process were evaluated 10 days after the application of scaffolds by microCT and histology, respectively. Similar to RA mice, diminished angiogenesis was observed at 10 dpf in callus tissue treated with the PCL scaffold without growth factors under systemic inflammation in vivo. In contrast, the PCL scaffold with SPP1 and/or CXCL12 induced a significant angiogenic response in calluses at 10 dpf, resulting in the formation of more blood vessels under RA conditions (Fig. S8A, B and Fig. 6b, c). Consistent with the observation of in vitro angiogenesis assays, SPP1 and CXCL12 in combination achieved the greatest restorative effect on blood vessel formation in RA mice compared to the individual SPP1 and CXCL12 treatments. We also performed IHC to detect endomucin-positive blood vessels in fracture calluses at 10 dpf and revealed significantly more blood vessels in the fracture calluses of RA mice to which the PCL scaffold containing both SPP1 and CXCL12 was applied (Fig. 6d, e). More importantly, coincident with the restoration of blood vessels in the fracture callus, newly formed woven bone was also observed in RA mice treated with SPP1 and CXCL12 via the PCL scaffold. Individual SPP1 and CXCL12 treatment similarly increased the amount of woven bone (eightfold increase) in the region adjacent to the fracture at 10 dpf (Fig. S8C, D). The application of SPP1 and CXCL12 together increased woven bone in calluses at 10 dpf by 25-fold. Notably, the newly formed bone replaced the fibrotic tissue and unified the fracture nonunion in the RA mice after 10 days of local treatment with SPP1 and CXCL12 (Fig. 6f, g). Finally, we measured the bone biomechanical properties by the torsion test and found that the maximum bone strength in the RA mice treated with SPP1 and CXCL12 was significantly restored by 28 dpf (Fig. 6h). Hence, these findings strongly suggest that the local release of SPP1 and CXCL12 via PCL scaffolds represents an effective therapeutic approach to treat impaired angiogenesis and fracture nonunion under inflammatory conditions.

## Discussion

Despite the growing knowledge of atrophic nonunion from animal models, fracture nonunion remains an exceedingly challenging clinical problem with limited and mainly invasive therapeutic interventions. To date, the atrophic nonunion models^64^ available for mechanistic and therapeutic studies primarily rely on critical size bone defects and removal of the periosteum,^65^ which are particularly valuable to delineate the effect of periosteum tissue and progenitor cell differentiation on disease initiation and progression. Nevertheless, these animal models lack clinical relevance and rarely reflect the clinical scenario, since atrophic nonunion is more prevalent in patients under chronic inflammatory conditions, including those in diabetes and RA. In this study, we have demonstrated for the first time that RA mice developed atrophic nonunion under chronic inflammation induced by K/BxN serum. Specifically, the use of NF-κB reporter mice confirmed that elevated inflammation was observed in the RA mice, particularly in the hinder limbs, and maintained for at least 15 d. The expression of inflammatory factors was also induced locally in the fracture calluses of RA mice. Furthermore, the RA mice displayed (a) no fracture callus formation, (b) fibrotic scar tissue within the fracture, (c) diminished angiogenesis, and (d) poor mechanical performance, all of which are consistent with the clinical manifestations of atrophic nonunion in patients. Thus, this work highlights a novel RA nonunion model with high clinical relevance and provides a useful tool to study the pathology of atrophic nonunion under inflammation.

Although bone healing failure is due to interplay between multiple components, including defects of growth factors, progenitor cells and mechanical factors, a lack of blood supply has long been believed to be an essential trigger for fracture healing defects, particularly atrophic nonunion. Clinical studies over the past decade have revealed a threefold increase in the nonunion rate in patients with ischemic injuries to tibia fracture compared to average fracture patients.^66^ More importantly, fracture nonunion can be significantly improved by reconstruction of the vascular structure and restoration of blood supply,^67^ suggesting the pivotal role of angiogenesis in the development of fracture nonunion. Recent studies have indicated that chondrocytes and osteoblasts are two major cell sources that secrete angiogenic factors to restore the blood supply via angiogenesis during the initial fracture repair process. Restoration of the blood supply then brings growth factors and oxygen to facilitate bony callus formation through chondrogenic and osteogenic differentiation of progenitor cells and brings osteoclasts to remodel the callus to regain the normal bone structure. Despite these findings, the underlying mechanism by which impaired angiogenesis results in atrophic nonunion, especially under pathological inflammatory conditions, remains largely unknown. Therefore, we comprehensively screened the angiogenic factors secreted from chondrocytes and osteoblasts under IL-1β treatment. Surprisingly, unlike chondrocytes, IL-1β induced the expression of angiogenic factors in osteoblasts. In addition, IL-1β significantly induced VEGF expression in both chondrocytes and osteoblasts. Several studies have demonstrated that VEGF and VEGF signaling are a potent angiogenic factor and pathway, respectively, that stimulate vessel formation and fracture repair in mice.^57,68^ However, our in vivo finding demonstrated that the RA mice developed impaired angiogenesis due to inflammation, which led us to speculate that the reduced angiogenic factors, but not VEGF, are likely the direct downstream targets that mediate the reduced angiogenesis observed in vivo. More importantly, SPP1 and CXCL12 were identified as the two angiogenic factors reduced to the greatest extent in IL-1β-treated chondrocytes. Their physiologic importance was evident in HUVEC experiments that showed that the addition of SPP1 and CXCL12 restored angiogenic defects present in supernatants harvested from IL-1b-treated chondrocyte cultures. These findings indicate that chondrocytes are the target cells that mediate the angiogenic defect observed in RA mice and that SPP1 and CXCL12 are potential downstream targets of inflammation in chondrocytes. However, our work does not completely dismiss the contribution of osteoblasts and other angiogenic factors to angiogenesis and atrophic nonunion.

This work uncovers several key findings that highlight its important translational implication to treat atrophic nonunion. Through the screening of angiogenic factors, in vitro angiogenesis assays and a unique RA fracture nonunion model, we have demonstrated that inflammation reduces the expression of SPP1 and CXCL12 in chondrocytes and leads to diminished angiogenesis and atrophic nonunion in mice. We also show that SPP1 and CXCL12 are critical downstream targets of inflammation and that supplementation with SPP1 and CXCL12 restored angiogenic capacity in vitro. More importantly, controlled release of SPP1 and CXCL12 locally via the PCL scaffold restored angiogenesis and fracture repair in RA mice. Therefore, this work provides a potential therapeutic approach to treat impaired angiogenesis and fracture nonunion in patients, especially under inflammatory conditions.

SPP1 and CXCL12 are chemokines demonstrated to stimulate angiogenesis both during normal organ development and under pathological conditions, such as various cancers. SPP1 itself can recruit endothelial cells to form new blood vessels^69^ and can also attract macrophages and function synergistically with other cytokines derived from macrophages to promote angiogenesis.^70^ Similarly, CXCL12 recruits CXCR4-positive endothelial cells and facilitates angiogenesis.^71^ In regard to fracture healing, global knockout of either *Spp1*^50^ or *Cxcl12*^49^ alone was shown to lead to angiogenesis defects and impaired fracture healing in mice, indicating the positive role of SPP1 and CXCL12 in angiogenesis and fracture healing. Interestingly, there is increasing evidence that SPP1 and CXCL12 expression is upregulated in various tissues under inflammatory conditions. Particularly in RA patients, high concentrations of SPP1 and CXCL12 were detected in synovial fluid, and both SPP1 and CXCL12 were overexpressed in RA synovial cells,^72,73^ which in turn led to excessive blood vessel invasion and synovial joint destruction. However, in contrast to the responses to inflammatory stimuli from synovial cells, SPP1 and CXCL12 expression was specifically reduced in chondrocytes by IL-1β. In accordance with these in vitro observations, the expression of SPP1 and CXCL12 was also decreased in the callus in RA. These findings highlight supplementation with SPP1 and CXCL12 as a promising therapeutic approach to treat atrophic nonunion, particularly chronic inflammation-induced nonunion.

Finally, as proof-of-concept experiment to show that the administration of SPP1 and CXCL12 can restore angiogenesis and is beneficial for atrophic nonunion fracture healing, we applied a PCL scaffold with SPP1 and CXCL12 to the fracture site in RA mice. It is well established that excessive SPP1 and CXCL12 are associated with cancer angiogenesis, metastasis, and malignancy.^74,75^ In addition, excessive angiogenesis mediated by SPP1 and CXCL12 in the synovium may exacerbate joint destruction in RA patients. Therefore, to avoid potential side effects, we engineered a PCL scaffold for the local sustained release of SPP1 and CXCL12 in the fracture site. The PCL scaffold is an FDA-approved biodegradable material that has been used for bone regeneration in animals.^76,77^ The fabricated scaffolds are fibrous, mimicking the morphology of extracellular matrix in the periosteum tissue. Notably, the scaffolds have a Young’s modulus and tensile strength comparable to those of the periosteum tissue.^78^ As expected, treatment with SPP1 or CXCL12 alone showed beneficial effects on angiogenesis and new bone formation in the RA callus. More importantly, consistent with the in vitro findings, the combination of SPP1 and CXCL12 exerted a synergistic effect in vivo, i.e., significantly induced angiogenesis to a greater extent than either factor alone. Surprisingly, both factors together also induced bony callus formation and fracture union at 10 dpf in the RA mice. While the individual delivery of SPP1 or CXCL12 restored fracture repair in the RA mice with cartilage callus and areas of new bone formation at 10 dpf fracture, the combination treatment gave rise to a more mature bony callus without residual cartilage, suggesting accelerated fracture healing. This synergistic effect is likely due to the restoration of endomucin-positive blood vessels by SPP1 and CXCL12, given the evidence that endomucin-positive type H vessels are the most important vessels for facilitating fracture callus ossification and union.^79^ In addition, SPP1 can signal through multiple integrins to facilitate vessel formation through activation of the PI3K and MAP kinase pathways in endothelial cells.^80^ Similarly, CXCL12 can also activate the MAP kinase pathway through β-arrestin.^81^ Therefore, it is reasonable to speculate that SPP1 and CXCL12 synergistically induce angiogenesis in vitro and in vivo under inflammatory conditions through the activation of MAP kinase pathways in endothelial cells. Moreover, SPP1 is predominantly synthesized by osteoblasts;^43^ therefore, it is possible that increased vessel formation and bone formation are achieved through an SPP1-mediated positive feedback loop. CXCL12 can also induce osteoblast differentiation and mineralization,^82,83^ therefore accelerating bony callus formation in mice.

Overall, this work has high clinical relevance and significant translational potential. Our findings highlight the local delivery of SPP1 and CXCL12 as an important therapeutic option to improve angiogenesis and treat fracture atrophic nonunion, especially under inflammatory conditions. In this regard, further optimizing the release profiles of SPP1 and CXCL12 will be a focus of future work. In addition, it will be valuable to clarify the mechanism by which SPP1 and CXCL12 are affected by inflammation in a tissue-specific manner, particularly in chondrocytes under normal and disease conditions. This will be achieved by using inducible *Spp1* and *Cxcl12* loss-of-function and gain-of-function mice as well as “omics” high-throughput screening.

## Methods

### Animals

All animal studies were performed in accordance with approval of the Committees on Animal Resources at Washington University in St Louis. Male C57BL/6J wild-type mice were purchased from The Jackson Laboratory (#000664). *Relb-Luc* (NF-κB-GFP-Luciferase) reporter mice^84^ were purchased from The Jackson Laboratory (#027529) and used to visualize NF-κB activity in vivo. Systemic inflammation was induced in 12-week-old mice via i.p. injection of 100 μL of arthritic K/BxN serum at day 0 and day 3 and maintained by continuous injection every 5 d until the experimental endpoint. Phosphate-buffered saline (PBS) was administered to mice as a control. Bony fractures were generated on the right tibiae as previously described.^56^

### Bioluminescence imaging

PBS- and K/BxN serum-treated *Relb-Luc* reporter mice were given 150 mg·kg^−1^ luciferin and continuously monitored by an IVIS 50 imaging system (Xenogen/PerkinElmer) every 3 days for 15 days. The luminescence intensity of the right hind legs was analyzed by Living Image 3.0 software (Xenogen).

### Histological analyses of fracture calluses

The fractured tibiae were collected for histological analyses at 7, 10, 14, and 21 dpf. Following 10% neutral buffered formalin fixation and decalcification by 14% ethylenediaminetetraacetic acid, the fractured tibiae were embedded in paraffin, sectioned at 5 μm, and stained with Alcian blue/hematoxylin/orange G (ABH/OG) and tartrate-resistant acid phosphatase (TRAP). The cartilage and bone areas were measured using ImageJ software (Wayne Rasband) based on ABH/OG staining. The osteoclast surface per bone surface (Oc. S/BS) was measured and calculated based on TRAP staining of 14 dpf fracture calluses. IHC staining for type III collagen (1:500, Abcam, #ab7778), SPP1 (1:50, Abcam, #ab8448), and CXCL12 (1:50, LSBio, #LS-B2437) was performed via proteinase K antigen retrieval and DAB (3,3′-diaminobenzidine, Vector Laboratories, #SK-4100)-mediated colorimetric development. Immunofluorescence staining for endomucin (1:100, Santa Cruz, #sc-65495) was performed via proteinase K antigen retrieval and Alexa Fluor 488 antibody labeling kit (Thermo Fisher, #A20181)-mediated fluorescent development. Quantification of the vessel number was based on endomucin immunofluorescence staining of the central fracture area (800 μm × 600 μm), and the relative vessel count was calculated by normalization of the experimental group to the control group.

### MicroCT analyses

The fractured tibiae were collected at 10, 14, and 21 dpf and examined by a microCT scanner (VivaCT 40, Scanco) with the following parameters: 55 kV, 145 μA, and a 300 ms integration time. 3D images were generated using Scanco software. Quantification of the BV and BV/TV was based on 600 slices centered on the fracture site as previously described.^56^ Relative BV and BV/TV were calculated by normalization of the experimental group to the control group.

### In vivo angiogenesis analysis

Each animal was perfused with Microfil MV-122 (Flowtech, #MV-122), a lead chromate-based contrast agent, after which the vascular structure surrounding the fracture site in the tibia was examined by microCT with the following parameters: 55 kV, 145 μA, and a 300 ms integration time. 3D images were generated using Scanco software. In this experiment, we analyzed the center of the fracture area where the atrophic region was located and the surrounding region. Quantification of the vessel number was based on 20 slices centered on the fracture site as previously described.^56^ The relative vessel count was calculated by normalization of the experimental group to the control group.

### Biomechanical torsion testing

The fractured tibiae were harvested at 28 dpf, and the ends were secured with methacrylate (MMA) in 1.2-cm-long cylinders to place the fracture site in the middle. The fracture tibiae were tested in terms of torsion using a custom LabVIEW (National Instruments) program until failure. The maximum torque and displacement at maximum torque were recorded and processed by a custom MATLAB 2017b program (Mathworks).

### Primary chondrocyte isolation and culture

Primary chondrocytes were isolated from the ribcages of neonatal C57BL/6J pups as previously described.^56^ Briefly, the ribcages were dissected without soft tissue, followed by 2 mg·mL^−1^ pronase (Millipore Sigma, #10165921001) and 3 mg·mL^−1^ collagenase D (Millipore Sigma, #11088866001) digestion with agitation. The remaining sterna were further digested with 3 mg·mL^−1^ collagenase D for 4 to 6 h. Chondrocytes were collected and cultured in DMEM with 10% FBS. Following 48 h of treatment with vehicle and 1 ng·mL^−1^ IL-1β, the culture medium was collected for the angiogenic protein array and HUVEC angiogenesis assay.

### Primary osteoblast isolation and culture

Primary osteoblasts were isolated from the calvarias of neonatal C57BL/6J pups as previously described.^85^ Briefly, after removing the soft tissue and sutures, calvarias were digested with 0.1% dispase (Millipore Sigma, #D4693) and 0.1% collagenase P (Millipore Sigma, #11249002001). Osteoblasts released from the mouse calvarias were collected and stimulated with 50 μg·mL^−1^ ascorbic acid (Millipore Sigma, #57803) for 3 days for maturation. Following the treatment of mature osteoblasts with vehicle and 1 ng·mL^−1^ IL-1β for 48 h, the culture medium was collected for the angiogenic protein array and HUVEC angiogenesis assay.

### Angiogenic protein array

A Proteome Profiler Mouse Angiogenesis Array Kit (R&D, #ARY015) was used to examine the expression of angiogenic factors in medium collected from chondrocyte and osteoblast cultures according to the manufacturer’s protocol. A Bio-Rad ChemiDoc imaging system was used to visualize and quantify the array signals.

### In vitro angiogenesis assay

In the tube formation assay, 4 × 10^4^ HUVECs were seeded and cultured in 96-well plates precoated with Matrigel (Corning, #356237) for 12 h. The numbers of formed tubes were examined by ImageJ software. In the migration assay, medium collected from chondrocyte cultures was added to the bottom chamber. HUVECs (4 × 10^4^) were seeded and cultured in the upper chambers of a 24-well Transwell plate (Millipore Sigma, #CLS3422) with serum-free medium for 12 h. Migrated HUVECs were counted based on crystal violet staining. Then, 100 ng·mL^−1^ CXCL12 (Roche, #460-SD-010) and 500 ng·mL^−1^ SPP1 (Roche, #441-OP-050) were individually added to chondrocyte culture medium for HUVEC tube formation and migration assays. HUVEC proliferation and apoptosis in culture medium from vehicle- and IL-1β-treated chondrocytes were assessed with a Roche Cell Proliferation ELISA Kit (Roche, #11647229001) and Cell Death Detection ELISA Kit (Roche, #11774425001), respectively.

### Fabrication of PCL scaffold with growth factors

The PCL scaffold was fabricated with PLGA microspheres containing SPP1 and CXCL12 via a coaxial electrospraying and electrospinning method as described previously.^63^ Briefly, PCL (Millipore Sigma, #440744) was dissolved in chloroform (Millipore Sigma, #372978) at a final concentration of 5% and stored in syringe pump A. A solution of 5% PLGA (Millipore Sigma, #P2191) was dissolved in methylene chloride (Millipore Sigma, # M1550000) and stored at syringe pump B. The growth factors 100 μg·mL^−1^ SPP1 (Peprotec, #120-35) and 20 μg·mL^−1^ CXCL12 (Peprotec, #300-28B) were mixed with 0.5% gelatin (Millipore Sigma, #G6650) in syringe pump C. PCL was injected at a rate of 5 mL·h^−1^ onto the collecting mandrel 20 cm away. The PCL solution was charged at +17 kV, while the collecting mandrel was charged at −10 kV. The diameter of the collecting mandrel was 13 cm, and the rotation rate was 800 r·min^−1^. The PLGA solution was sprayed at a rate of 1 mL·h^−1^ into the exterior aisle, and the solution containing growth factors was simultaneously sprayed at a rate of 0.5 mL·h^−1^ into the interior aisle. The inner diameter of the coaxial apparatus was 0.70 mm, and the outer diameter was 1.65 mm. The coaxial aisle was charged at +20 kV, while the collecting mandrel was charged at −10 kV. The distance between the electrospray needle and collector was 30 cm. Four types of scaffolds were fabricated: scaffolds with no growth factor, scaffolds with SPP1 alone, scaffolds with CXCL12 alone, and scaffolds with both SPP1 and CXCL12.

### Scaffold characterization

We mixed a PLGA solution with 10 μg·mL^−1^ rhodamine B (Millipore Sigma, #83689) and growth factor solution with 50 mg·mL^−1^ Hoechst (Millipore Sigma, #63493). The core–shell structure was examined with a Zeiss LSM880 confocal microscope. The scaffolds containing SPP1 and CXCL12 were sputter-coated with gold. The fiber structure and microsphere distribution were examined by SEM. The mechanical properties of the PCL scaffold were examined by tensile testing. Briefly, the PCL scaffold at a 150 μm thickness was cut into pieces 15 mm in length and 1 mm in width. A 100 N load cell and tensile displacement rate of 0.5 mm·min^−1^ were applied to the scaffold. The force and displacement were recorded. Tensile stress and strain were calculated. The Young’s modulus was determined in the elastic deformation region of the stress–strain curve.

### Release profiles of growth factors from PCL scaffolds

PCL scaffolds (50 mg) containing growth factors (SPP1 and CXCL12) were placed into 1 mL of PBS (HyClone, #SH30013.0.3) supplemented with 1% penicillin and streptomycin (Thermo Fisher, #15140122) and incubated at 37 °C for 4 weeks. The PBS was collected at predetermined time points, and an equal volume of fresh PBS was added. The collected PBS was used for ELISAs to determine the concentrations of SPP1 and CXCL12 according to the manufacturers’ protocol (R&D Systems, #DOST00 for SPP1; Peprotec, #900-M92 for CXCL12).

### In vitro bioactivity assay with PCL scaffolds

PCL scaffold was placed on the bottom of a 24-well plate. Five hundred microliters of collagen gel (Corning, #354236) was cast on the top of the scaffold, after which CM-Dil (Invitrogen, #C7000)-labeled HUVECs were seeded at a density of 1 × 10^5^ cells per mL. After 5 days of culture, the constructs were fixed with a 4% paraformaldehyde solution (Thermo Fisher, #AAJ19943K2). The cells were imaged with a confocal microscope. Z-stack images were taken at a 20 µm thickness. HUVEC migration and lumen formation were quantified based on the constructed 3D images.

### Real-time qPCR

A 4 mm fracture callus was isolated from control and RA mice and homogenized for RNA extraction using an RNeasy Mini Kit (Qiagen, #74106). cDNA synthesis and real-time qPCR were performed following the manufacturers’ protocol. The primer sequences for *Il1b*, *Il6*, *Il10*, *Tnfa*, *Spp1*, *Cxcl12*, and *Actb* are presented in Table 1.

**Table 1:** Primer sequences for qPCR

Gene	Sequences
*Il1b*	5′-GCA ACT GTT CCT GAA CTC AAC T-3′ 5′-ATC TTT TGG GGT CCG TCA ACT-3′
*Il6*	5′-TCC AGT TGC CTT CTT GGG AC-3′ 5′-GTA CTC CAG AAG ACC AGA GG-3′
*Il10*	5′-GCT CTT ACT GAC TGG CAT GAG-3′ 5′-CGC AGC TCT AGG AGC TAG TG-3′
*Tnfa*	5′-CAC ACT CAG ATC ATC TTC TCA A-3′ 5′-AGT AGA CAA GGT ACA ACC CAT C-3′
*Spp1*	5′-TCG TCA TCA TCG TCG TCC A-3′ 5′-AGA ATG CTG TGT CCT CTG AAG-3′
*Cxcl12*	5′-GAC TCA CAC CTC TCA CAT CTT G-3′ 5′-GTG CCC TTC AGA TTG TTG C-3′
*Actb*	5′-AGA TGT GGA TCA GCA AGC AG-3′ 5′-GCG CAA GTT AGG TTT TGT CA-3′

### Western blot analysis

Primary chondrocytes were seeded at a density of 1 × 10^6^ cells per well in a six-well plate and treated with vehicle, 20 ng·mL^−1^ IL-6 (R&D Systems, #406-ML-005) and 20 ng·mL^−1^ TNFα (R&D Systems, #410-MT-010) for 48 h. Cell lysates were separated by SDS-polyacrylamide gel electrophoresis and examined with antibodies against the following: SPP1 (1:1 000, Abcam, #ab8448), CXCL12 (1:1 000, LSBio, #LS-B2437) and β-actin (1:4 000, Sigma, #2228).

### Statistics

Statistical analyses were performed using GraphPad Prism. The two-tailed Student’s *t-*test was used to determine the significance between two groups. Comparisons among multiple groups were performed using two-way ANOVA followed by Tukey’s test. All data are presented as the mean ± SD of at least three independent experiments. *P* < 0.05 was used to indicate statistical significance.

## Supplementary Information

### Supplementary Information

Supplemental Figure 1

Supplemental Figure 2

Supplemental Figure 3

Supplemental Figure 4

Supplemental Figure 5

Supplemental Figure 6

Supplemental Figure 7

Supplemental Figure 8

Supplementary Information

### Supplementary information
