# Supplementary figures and images for: Targeting angiogenesis for fracture nonunion treatment in inflammatory disease
Source: Bone Res. 2021 Jun 7;9:29. doi: 10.1038/s41413-021-00150-4 (PMC8184936; doi:10.1038/s41413-021-00150-4)

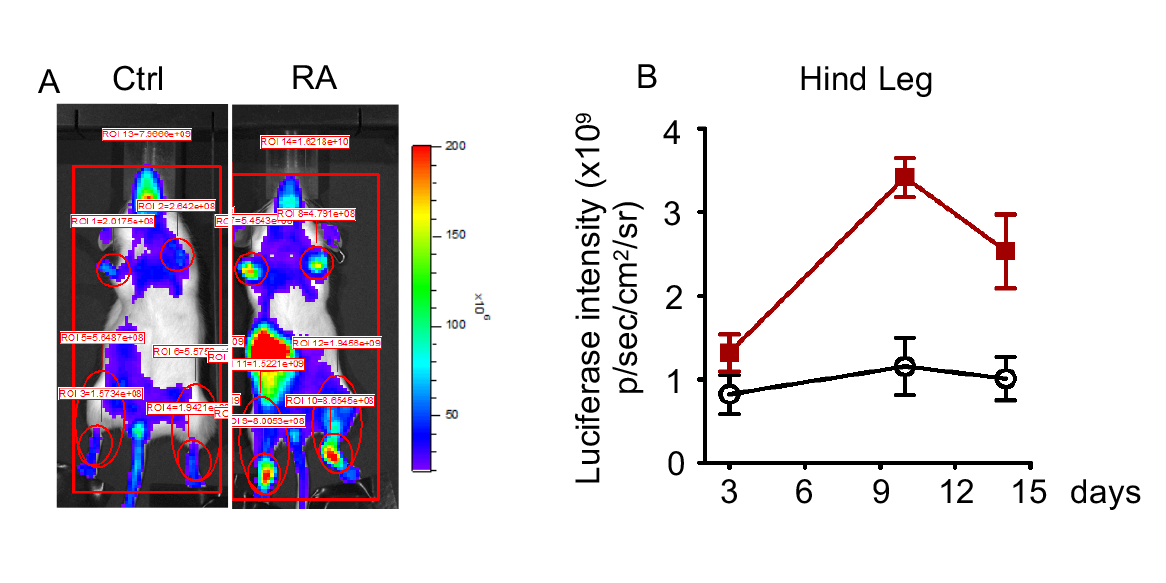

Supplement: Supplementary file 1 — Supplemental Figure 1 [file 41413_2021_150_MOESM1_ESM.tif]

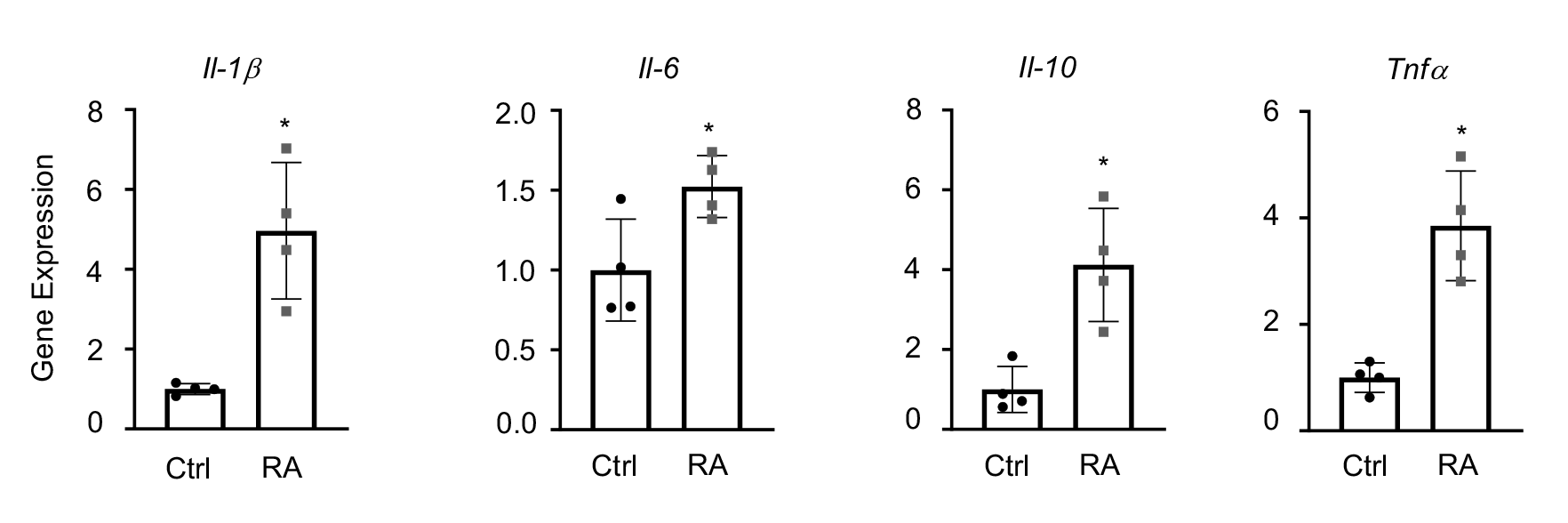

Supplement: Supplementary file 2 — Supplemental Figure 2 [file 41413_2021_150_MOESM2_ESM.tif]

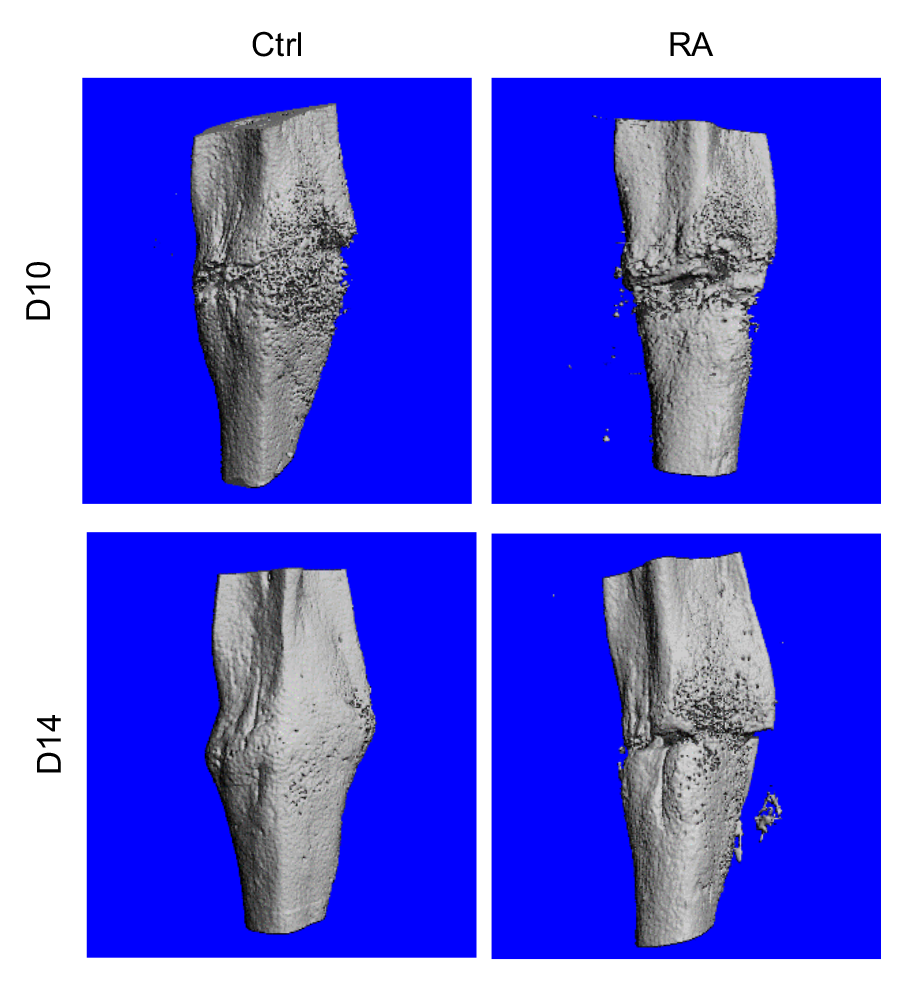

Supplement: Supplementary file 3 — Supplemental Figure 3 [file 41413_2021_150_MOESM3_ESM.tif]

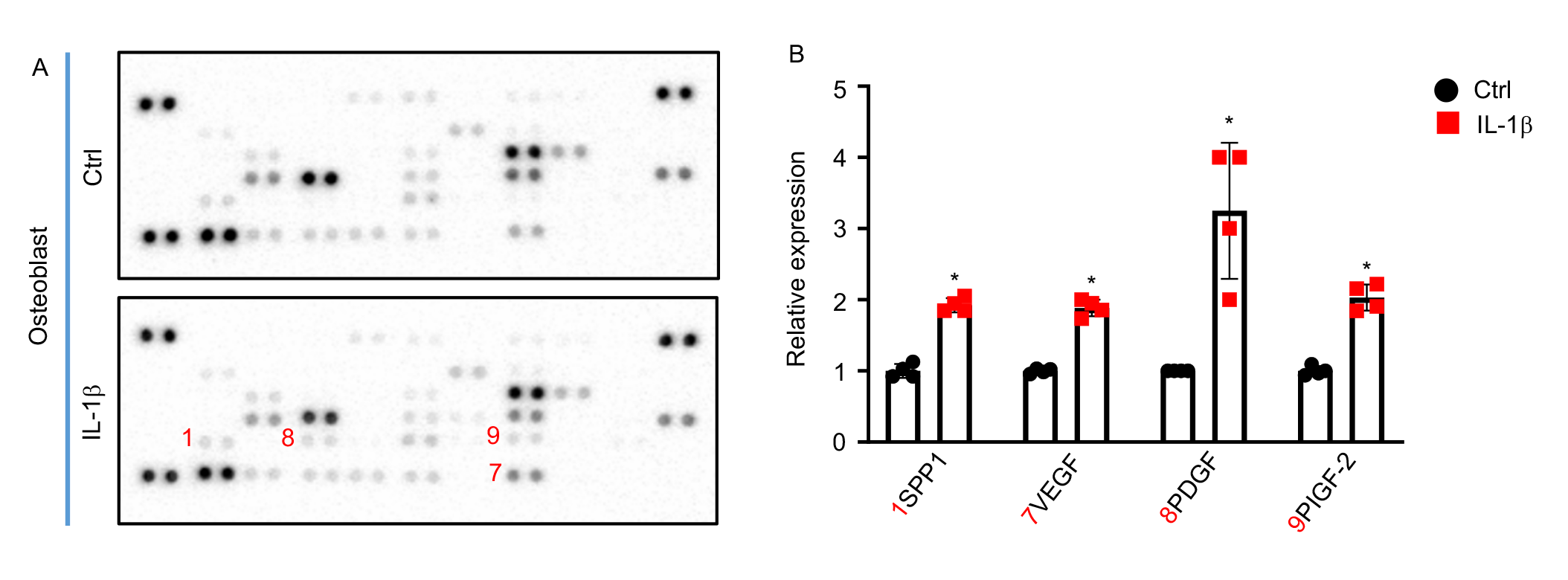

Supplement: Supplementary file 4 — Supplemental Figure 4 [file 41413_2021_150_MOESM4_ESM.tif]

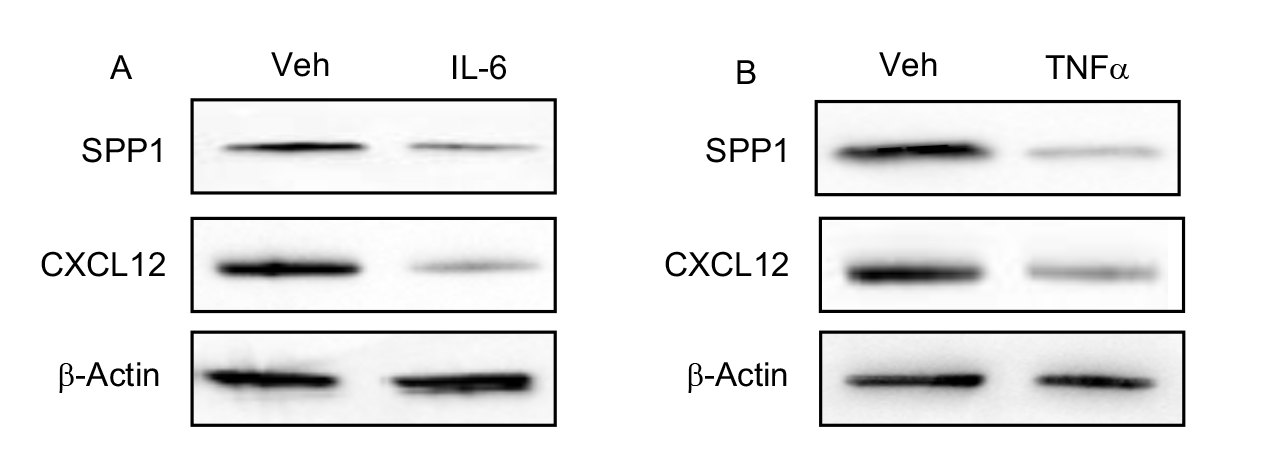

Supplement: Supplementary file 5 — Supplemental Figure 5 [file 41413_2021_150_MOESM5_ESM.tif]

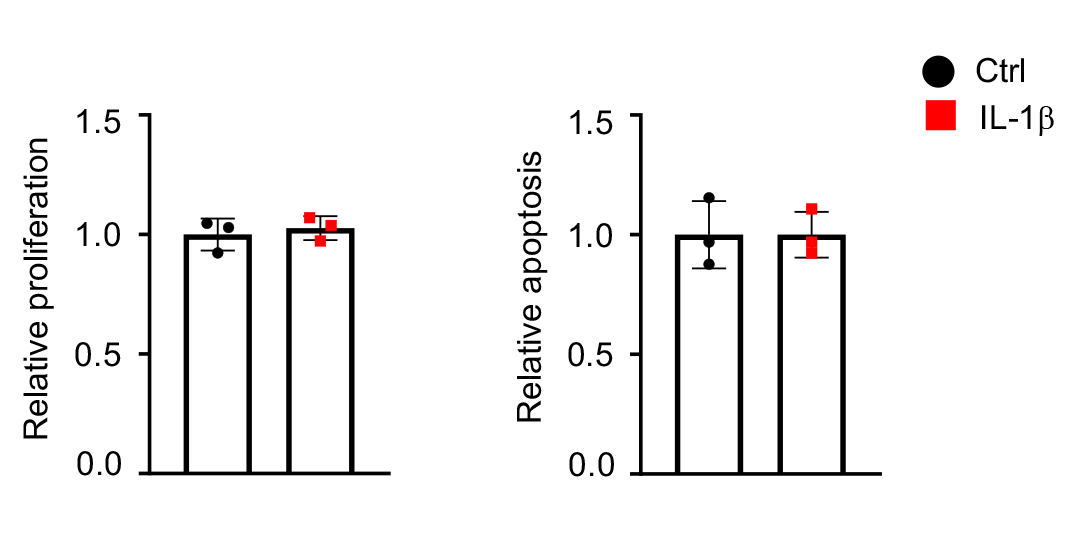

Supplement: Supplementary file 6 — Supplemental Figure 6 [file 41413_2021_150_MOESM6_ESM.tif]

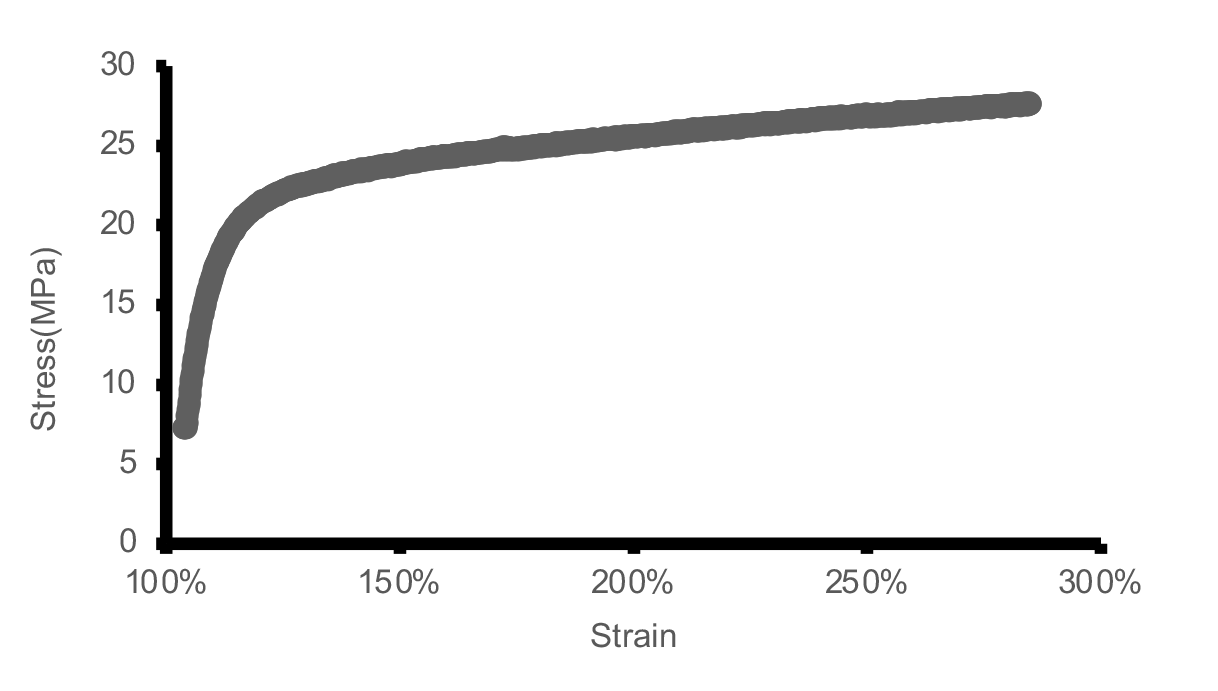

Supplement: Supplementary file 7 — Supplemental Figure 7 [file 41413_2021_150_MOESM7_ESM.tif]

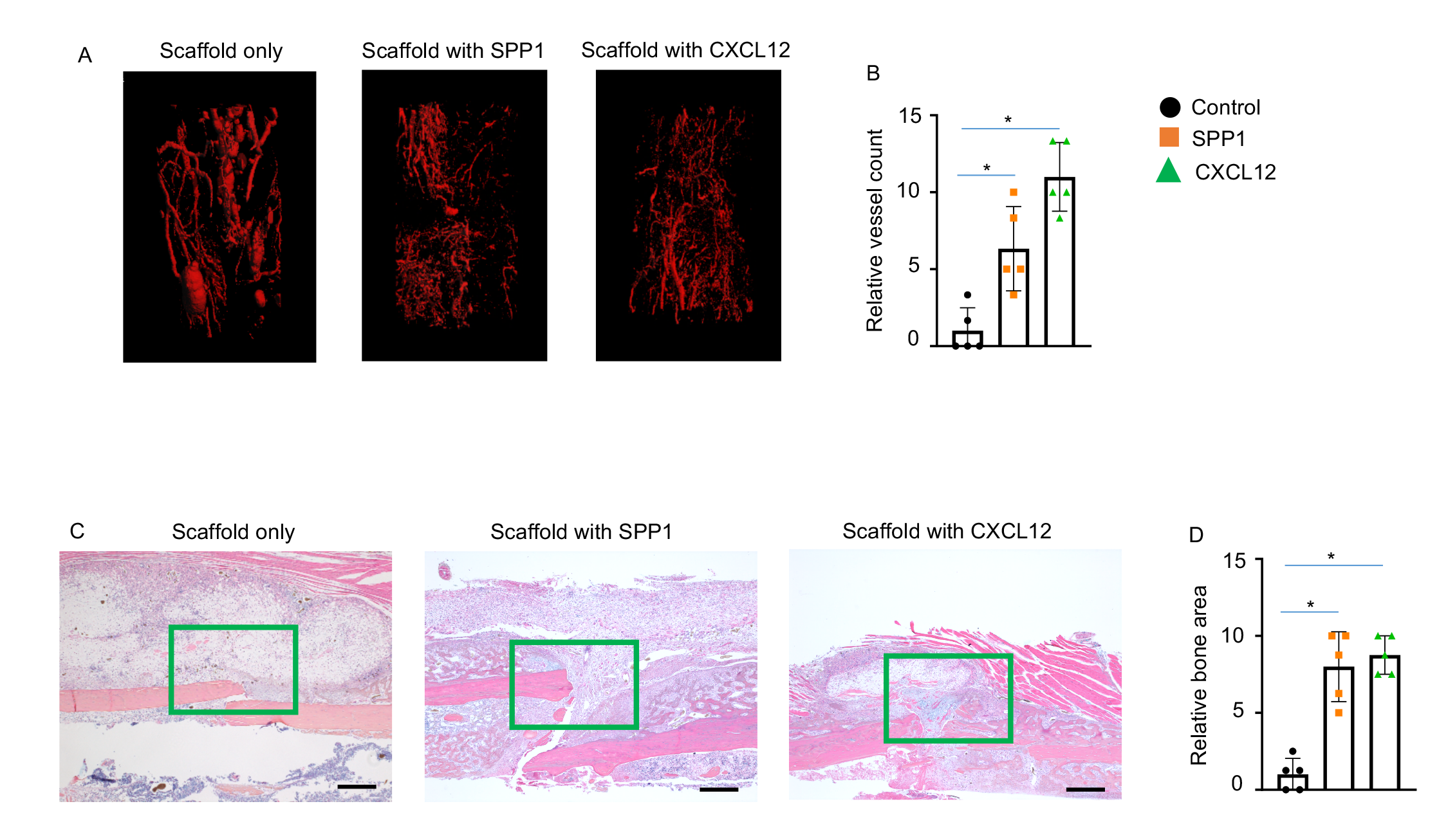

Supplement: Supplementary file 8 — Supplemental Figure 8 [file 41413_2021_150_MOESM8_ESM.tif]
