# Supplementary material for: Targeting angiogenesis for fracture nonunion treatment in inflammatory disease
Source: Bone Res. 2021 Jun 7;9:29. doi: 10.1038/s41413-021-00150-4 (PMC8184936; doi:10.1038/s41413-021-00150-4)
Supplement: Supplementary file 9 — Supplementary Information [file 41413_2021_150_MOESM9_ESM.docx]

**Figure legends**

**Fig. S1 Elevated NF-κB activity in the lower limbs of RA mice.** (A) *Relb-Luc* reporter mice were used to visualize NF-κB activity *in vivo* following PBS (Ctrl) and K/BxN (RA) administration. (B) Quantification of luciferase intensity in the lower limbs of *Relb-Luc* reporter mice at the indicated time points (n=3). **p*< 0.05 compared with control by Student’s *t-*test.

**Fig. S2 Increased expression of inflammatory cytokines in RA fracture calluses.** Real-time qPCR analyses were performed to determine the relative expression of *Il1b*, *Il6*, *Il10* and *Tnfa* in fracture calluses at 7 dpf from the control and RA mice (n=4). The mRNA levels were normalized to that of *Actb* and then normalized to the control group. **p*< 0.05 compared with control by Student’s *t-*test.

**Fig. S3 Systemic inflammation led to reduced bone formation in RA mice.** MicroCT assessment of mineralized bone within fracture calluses at 10 and 14 dpf from the control and RA mice (n=5).

**Fig. S4 Inflammation led to the upregulation of angiogenic factors in osteoblasts.** (A) Representative images of an angiogenesis proteasome array from the culture medium of osteoblasts following vehicle and IL-1β treatment. (B) Quantification of blot intensity from the angiogenesis proteasome array (n=4). All results were normalized to the controls. **p*< 0.05 compared with control by Student’s *t-*test.

**Fig. S5 IL-6 and TNFα treatment led to the downregulation of SPP1 and CXCL12 in chondrocytes.** Primary chondrocytes were treated with IL-6 and TNFα for 48 hours, and the protein levels of (A) SPP1 and (B) CXCL12 were examined by western blot analysis.

**Fig. S6 Conditioned media did not affect the proliferation or apoptosis of HUVECs.** Quantification of **the** cell proliferation and apoptosis of HUVECs cultured in medium collected from vehicle- and IL-1β treated chondrocytes. All results were normalized to the controls. **p*< 0.05 compared with control by Student's *t*-test.

**Fig. S7 Tensile stress and strain of PCL scaffolds.**

**Fig. S8 Supplementation with SPP1 or CXCL12 partially restored angiogenesis and bone formation in RA mice.** (A) MicroCT assessment of newly formed vessels within fracture calluses from RA mice treated with scaffold loaded with SPP1 or CXCL12 (n=5) at 10 dpf. (B) Quantification of the vessels in RA fracture calluses (n=5) at 10 dpf based on microCT assessment. The results were normalized to the scaffold only group. **p*< 0.05 compared with control by two-way ANOVA. (C) Representative ABH/OG staining of fracture callus sections from RA mice treated with scaffold loaded with SPP1 or CXCL12 at 10 dpf (n=5). (D) Histomorphometric quantification of the bone area was performed on 10 dpf fracture callus sections from RA mice treated with scaffold loaded with SPP1 or CXCL12 (n=5). The results were normalized to the scaffold group. **p*< 0.05 compared with control by two-way ANOVA. Scale bar, 200 μm.
